# Supplementary material for: Entropy-Driven Heterogeneous Crystallization of Hard-Sphere Chains under Unidimensional Confinement
Source: Polymers (Basel). 2021 Apr 21;13(9):1352. doi: 10.3390/polym13091352 (PMC8122411; doi:10.3390/polym13091352)
Supplement: Supplementary file 1 [file polymers-13-01352-s001.zip › Fig13a.pdf]

N = 100 at 0.60 (bottom layer), after 2x10^7 MC steps

This area requires a 3D PDF enabled viewer such as Adobe Reader.
