# Supplementary material for: Entropy-Driven Heterogeneous Crystallization of Hard-Sphere Chains under Unidimensional Confinement
Source: Polymers (Basel). 2021 Apr 21;13(9):1352. doi: 10.3390/polym13091352 (PMC8122411; doi:10.3390/polym13091352)
Supplement: Supplementary file 1 [file polymers-13-01352-s001.zip › Polymers-1171645_interactive 3D version.pdf]

polymers

Article

Entropy-Driven Heterogeneous Crystallization of Hard-Sphere Chains under Unidimensional Confinement

Pablo Miguel Ramos, Miguel Herranz, Katerina Foteinopoulou, Nikos Ch. Karayiannis * and Manuel Laso

Institute for Optoelectronic Systems and Microtechnology (ISOM) and Escuela Técnica Superior de Ingenieros Industriales (ETSII), Universidad Politécnica de Madrid (UPM), José Gutierrez Abascal 2, 28006 Madrid, Spain; pm.ramos@alumnos.upm.es (P.M.R.); miguel.herranzf@upm.es (M.H.); kfoteinopoulou@etsii.upm.es (K.F.); mlaso@etsii.upm.es (M.L.)
* Correspondence: nkarayiannis@etsii.upm.es; Tel.: +34-910-677-318

Abstract: We investigate, through Monte Carlo simulations, the heterogeneous crystallization of linear chains of tangent hard spheres under confinement in one dimension. Confinement is realized through flat, impenetrable, and parallel walls. A wide range of systems is studied with respect to their average chain lengths ($N = 12$ to 100) and packing densities ($\varphi = 0.50$ to 0.61). The local structure is quantified through the Characteristic Crystallographic Element (CCE) norm descriptor. Here, we split the phenomenon into the bulk crystallization, far from the walls, and the projected surface crystallization in layers adjacent to the confining surfaces. Once a critical volume fraction is met, the chains show a phase transition, starting from regions near the hard walls. The established crystal morphologies consist of alternating hexagonal close-packed or face-centered cubic layers with a stacking direction perpendicular to the confining walls. Crystal layer perfection is observed with an increasing concentration. As in the case of the unconstrained phase transition of athermal polymers at high densities, crystal nucleation and growth compete with the formation of sites of a fivefold local symmetry. While surface crystallites show perfection with a predominantly triangular character, the morphologies of square crystals or of a mixed type are also formed. The simulation results show that the rate of perfection of the surface crystallization is not significantly faster than that of the bulk crystallization.

Keywords: monte carlo; molecular simulation; crystallization; hard sphere; athermal chain; polymer; confinement; packing; hexagonal close-packed; face-centered cubic; entropy-driven; phase transition; triangular crystal; square crystal; fivefold

Citation: Ramos, P.M.; Herranz, M.; Foteinopoulou, K.; Karayiannis, N.C.; Laso, M. Entropy-Driven Heterogeneous Crystallization of Hard-Sphere Chains under Unidimensional Confinement. *Polymers* **2021**, *13*, 1352. https://doi.org/10.3390/polym13091352

Academic Editors: Sasaki Takashi and Jens-Uwe Sommer

Received: 20 March 2021
Accepted: 19 April 2021
Published: 21 April 2021

Publisher's Note: MDPI stays neutral with regard to jurisdictional claims in published maps and institutional affiliations.

Copyright: © 2021 by the authors. Licensee MDPI, Basel, Switzerland. This article is an open access article distributed under the terms and conditions of the Creative Commons Attribution (CC BY) license (http://creativecommons.org/licenses/by/4.0/).

1. Introduction

The phase and morphology of a system dictate its macroscopic properties. Thus, understanding crystal nucleation and growth as a function of processing history and conditions could lead to the design of novel materials with improved properties. While crystallization has been exhaustively studied through experiments, theory, and simulations, a lot of its aspects remain poorly understood, especially at the level of atoms and molecules. The complexity of the phenomenon increases appreciably when interfacial or surface effects and spatial heterogeneity are considered, beyond the reference bulk case [1–4]. Further intricacy is added by constraints imposed by inherent chain connectivity, as in the case of macromolecular systems, which are additionally characterized by a wide range of characteristic time and length scales, the global ones being dominated by the presence of entanglements [5–11].

Early and pioneering molecular dynamics simulations have unmistakably shown that hard sphere systems crystallize [12]. For such phase transition to take place, the two critical conditions are: (i) a critical volume fraction is reached; and (ii) the evolution of the system is tracked for a sufficiently long time [13]. In a pattern analogous to bulk athermal

monomers, random packings of linear and fully flexible chains of tangent hard spheres are entropically driven to the crystal phase at sufficiently high volume fractions [14]. Crystal morphologies of athermal polymers adopt random hexagonal close-packed layers of hexagonal close-packed and face-centered cubic characters. These alternating layers possess a unique stacking direction and are free of twin defects, as the latter are incompatible with the entropic barriers imposed by chain connectivity [15]. The critical phase transition of hard-sphere chains is heavily affected by factors, such as bond gaps [16,17] and/or chain stiffness [18].

The crystallization or solidification of hard-sphere chains in the bulk has been reported in the literature, considering as the influence of chain length, backbone stiffness, and bond geometry [14,16–22], and highlighting similarities and differences with respect to the crystal nucleation and growth of monomeric analogs [13,23–29]. The jamming or phase transition of athermal, initially random packings, both of monomers and of chains, under confinement have not been studied as extensively as the bulk case, but independent works have provided significant insights over the years [3,30–42].

Reviews summarizing the advances achieved through experimental and simulation studies on general atomic crystallization in confinement of a variety of systems and confining agents, including droplets, parallel plates, microemulsions, vesicles, pores, wedges, patterned, and nano-fabricated environments, can be found in [43,44]. Confinement can profoundly affect the crystallization mechanism, the location of the freezing and melting transitions, as well as the established ordered morphologies, compared to the bulk, unconstrainted case. The behavior of the hard sphere model and the corresponding packing properties can be effectively studied experimentally through suspensions of colloidal spheres or granular materials [44,45]. Such realizations, by being characterized by macroscopic time and length scales, allow not only for the experimental determination of their structure and dynamics, but also the visualization of their crystal morphologies. For example, the buckling mechanism of micro-sized colloidal suspensions, mimicking hard-sphere packings, in wedge geometries has been studied in [46,47].

In the present contribution, we conduct extensive Monte Carlo (MC) simulations based on local and chain-connectivity-altering algorithms [48,49] on fully flexible, linear chains of tangent hard spheres under unidimensional confinement. The latter is achieved through flat, parallel, and impenetrable walls in one dimension. We study the effect of the packing density, φ, average chain length, N, and inter-wall distance, d_{wall}, on the ability of chains to crystallize. The local environment around each site is analyzed through the characteristic crystallographic element (CCE) norm descriptor [50], which has recently been generalized to analyze 3-D and 2-D systems with respect to reference crystal templates [51]. We describe the heterogeneous crystal nucleation and growth through two distinct contributions: the bulk crystallization far from the confining surfaces and the surface crystallization for the top and bottom layers adjacent to the walls. Perfectly ordered 2-D crystal structures are formed on the wall layers of a predominantly triangular (TRI) character. Close-packed crystallites grow near the walls, where the local density is high, and, for sufficiently high packing densities, propagate to the inner regions of the system, until crystal uniformity is achieved. Bulk crystals consist of layers with a stacking direction perpendicular to the walls with a primarily unique face-centered cubic (FCC) or hexagonal close-packed (HCP) similarity. The formation of a fivefold local symmetry is again seen as acting antagonistically against crystal nucleation and growth. The final ordered structures of layered crystals are free of defects in the form of twinning.

Section 2 presents the molecular model, the simulation method, and the systems studied, while Section 3 hosts a brief analysis on how the local order on the surface and in the bulk is analyzed through the CCE norm descriptor [50,51]. The simulation results are presented in Section 4, a short discussion is provided in Section 5, and the manuscript is concluded, with the listing of the main findings in Section 6.

2. Molecular Model, Simulation Method, and Systems Studied

The hard sphere (HS) model has been adopted to describe all interactions between chain monomers, all of which are identical spheres with diameter σ. According to the HS model, the pair-wise energy, $u(r_{ij})$, is given by:

$$u(r_{ij}) = \begin{cases} 0, r_{ij} \geq \sigma \\ \infty, r_{ij} < \sigma \end{cases} \quad (1)$$

where r_{ij} is the distance between the centers of spheres i and j.

Linear chains follow the freely-jointed model. The bond length is equal to the sphere diameter within a tight tolerance of approximately 5×10^{-4}, thus leading to a tangency between bonded atoms. As there is no bending or torsional potential, the corresponding angles are allowed to fluctuate freely, subject only to constraints imposed by the excluded volume interactions.

For bulk systems with periodic boundary conditions (PBCs) applied on all dimensions, the packing density (volume fraction), φ, is defined as the volume occupied by the n spheres divided by the total volume of the simulation cell, V_{cell}:

$$\varphi = \frac{\pi n}{6 V_{\text{cell}}} \quad (2)$$

Confinement is realized here through the presence of flat, parallel, and impenetrable walls in one dimension. In the continuation and throughout the manuscript, we will consider x as the dimension of spatial restriction, i.e., the walls are parallel to this axis. No other interactions exist between the walls and the chain monomers except the non-overlapping condition, according to which:

$$u_{\text{wall}}(r_i) = \begin{cases} 0, r_i \geq \sigma/2 \\ \infty, r_i < \sigma/2 \end{cases} \quad (3)$$

where r_i is the distance between the center of sphere i and the closest wall in the confined dimension, and u_{wall} is the corresponding wall-monomer energy. The presence of the impenetrable walls causes a reduction in the volume available to pack the spherical monomers. Accordingly, we define a packing density that accounts for the inaccessible volume due to the confining agents, φ_{wall}, as:

$$\varphi_{\text{wall}} = \frac{\pi n}{6\,(d_{\text{wall}}(x) - 1)\,A_{yz}} \quad (4)$$

where $d_{\text{wall}}(x)$ is the distance between the parallel walls in the x dimension, measured in units of σ, and A_{yz} is the area of the simulation cell in the two unrestricted dimensions.

All systems to be presented below have been generated and equilibrated with the latest version of the Monte Carlo (MC) scheme, as analyzed in [48,49,52], based on chain-connectivity-altering moves, as introduced for atomistic systems [53,54]. First, the initial configurations at very dilute conditions (φ = 0.01) are borrowed from bulk systems with the same characteristics (chain length and total number of hard spheres). Then, all chains are fully unwrapped in space, and walls are inserted in the system along the confined dimension, creating a cubic simulation cell at a very low concentration. The system box is isotropically compressed through MC simulations, combining conventional volume shrinkage [49] and the wall-displacement algorithm [48], until the desired density is reached. Then, very long equilibration simulations are conducted under a constant volume. In both steps of system generation and equilibration, the MC suite consists of the following moves: flip (34.8%), rotation (15%), reptation (10%), intermolecular reptation (25%), configurational bias (15%), simplified end-bridging (sEB) (0.1%), and simplified intramolecular end-bridging (sIEB) (0.1%), where the number in parenthesis denotes the attempt probability. In the phase of generation, cell compressions are attempted every 1000 constant-volume moves, while the wall-displacement algorithm is automatically per-

formed, when applicable, as explained in [48]. All local MC moves are executed in a configurational bias pattern, with the number of trials depending strongly on the volume fraction [49].

The presence of chain-connectivity-altering moves requires and imposes polydispersity in chain lengths. These are allowed to fluctuate uniformly in the interval [N_{av} (1 − Δ), N_{av} (1 + Δ)], where N_{av} is the average chain length, and Δ is the half-width of the distribution divided by N_{av}. The four systems simulated in the present work are: 100-chains of N_{av} = 12, 50-chains of N_{av} = 24, 96-chains of N_{av} = 50, and 48-chains of N_{av} = 100, with Δ = 0.5 in all cases. The former (N = 12 and 24) and the latter (N = 50 and 100) pairs of systems consist of the same number of interacting sites, i.e., 1200 and 4800, respectively, allowing for further study of the effect of inter-wall distance in addition to chain length dependence.

For all systems, equilibrium simulations are conducted at packing densities that correspond to φ = 0.50, 0.52, 0.54, 0.56, 0.58, 0.60, and 0.61. In the past, we have demonstrated that freely-jointed chains of tangent hard spheres in the bulk crystallize once a volume fraction of [14,20,21] $\varphi_{bulk}^{crys} \approx 0.58$ is reached. This threshold for the phase transition shifts to lower values once bond gaps are introduced [16], eventually approaching that of monomeric hard sphere packings [12,13,23,28,55,56]. For the two different system sizes of n = 1200 (N_{av} = 12 and 24) and 4800 (N_{av} = 50 and 100) studied here Table 1 lists the inter-wall distance $d_{wall}(x)$ and the reduced packing densities due to the presence of the impenetrable walls, as calculated from Equation (4) as a function of the volume fraction.

Table 1. Packing density, φ, inter-wall distance, d_{wall}, and reduced packing density, φ_{wall}, for the systems of n = 1200 (N = 12 and 24) and 4800 (N = 50 and 100) interacting spheres.

φ	**0.50**	**0.52**	**0.54**	**0.56**	**0.58**	**0.60**	**0.61**
d_{wall} (n = 1200)	10.79	10.65	10.52	10.39	10.27	10.16	10.10
d_{wall} (n = 4800)	17.13	16.91	16.70	16.50	16.30	16.12	16.03
φ_{wall} (n = 1200)	0.551	0.574	0.596	0.620	0.643	0.665	0.677
φ_{wall} (n = 4800)	0.531	0.552	0.574	0.596	0.618	0.640	0.651

Using the nomenclature introduced in [48], for all systems studied here, the bond gaps are null ($dl \to 0$), while the number of confined dimensions, d_{conf}, and the cell aspect ratio, ζ, are both equal to unity (d_{conf} = 1; ζ = 1). The system configurations (frames) are recorded every 10^7 and 2×10^7 MC steps for the n = 1200 and 4800 systems, respectively. The total simulation time ranges from 5×10^{11} MC steps at the lowest packing density to 2×10^{12} MC steps at the highest one.

3. Analysis of the Local Structure

The local structure of the computer-generated configurations is analyzed through the latest version of the Characteristic Crystallographic Element (CCE) norm descriptor [51], which is able to detect the radial and orientational similarities of a local environment with respect to a given reference crystal X. The CCE norm functions on the basis that each crystal is identified by a unique set of geometric elements of symmetry and corresponding actions, which serve as a distinctive crystallographic fingerprint. Through a process of applying proper group symmetry elements, which is explained in detail in [51], every site (atom or particle), i, of the system is assigned an X-CCE norm value, ε_i^X, with respect to a reference crystal X. The closer this value is to zero, the higher the similarity of the local environment to this ideal crystal X. Once the value of the CCE norm is below a specific threshold, ε^{thres}, the site i is considered to be of X-similarity. By construction, the CCE norm method is highly discriminating: the value of the norms cannot be simultaneously very

low with respect to two different crystals. As in past simulations on non-overlapping spheres [16,51,52], the CCE-norm threshold adopts here a value of $\varepsilon^{\text{thres}} \leq 0.245$, independently of the reference crystal and its dimensionality.

The CCE norm descriptor is able to quantify structural similarity with respect to the following reference crystals: hexagonal close-packed (HCP), face-centered cubic (FCC), hexagonal (HEX), and body-centered cubic (BCC) in 3-D; triangular (TRI), square (SQU), and honeycomb (HON) in 2-D. Additionally, the 3-D fivefold (FIV) and 2-D pentagonal (PEN) local symmetries, which act as competitors to crystal formation in dense hard-sphere packings [55,56], are examined as well. Any atom that does not show similarity to any of the reference 3-D or 2-D crystals (or local symmetries) is labelled as amorphous (AMO) or more precisely as "unidentified" (or "none of the above").

Once the CCE norms are calculated over all reference crystals and over all atoms or particles of the system, an order parameter can be further calculated for the reference crystal X, S^X ($S^X \in [0,1]$):

$$S^X = \int_0^{\varepsilon^{\text{thres}}} P(\varepsilon^X)\mathrm{d}\varepsilon^X \quad (5)$$

where $P(\varepsilon^X)$ is the probability distribution function of the X-CCE norm.

For the general problem of crystallization under hard-wall confinement in any dimension ($d_{\text{conf}} \in [1,3]$), we divide the structural analysis into two distinct contributions: the bulk and the surface crystallinity. The latter corresponds to all atoms that belong to the top and bottom layers of the simulation cell or, in other words, to the spheres that lie the closest to the confining walls. For this, we create 2-D projections of the top and bottom layers consisting of these atoms and employ the CCE norm descriptor with respect to the TRI, SQU, and HON crystals and PEN local structure. For all other atoms ("bulk volume"), we employ the 3-D CCE norm quantifying similarity with respect to the HCP, FCC, HEX, BCC, and FIV local structures. Due to the nature of the interactions and the high packing density, HEX and BCC (in 3-D) and HON (in 2-D) crystallites do not appear in appreciable quantities in the present computer-generated systems. Consequently, in the continuation, we do not make a reference to these crystal structures.

Based on the above, a total degree of ordering, or total crystallinity, τ^c, can be calculated as the sum of the bulk, τ^{bulk}, and surface, τ^{surf}, ones:

$$\tau^{\text{c}} = \tau^{\text{bulk}} + \tau^{\text{surf}} = \sum_{l=1}^{N_{S,3}} S^{l,3} + \sum_{k=1}^{d_{\text{conf}}} \sum_{j=1}^{2} \sum_{i=1}^{N_{S,2}} S^{i,2} \quad (6)$$

where $N_{s,3}$ and $N_{s,2}$ are the numbers of reference X crystals in 3-D and 2-D, respectively. In Equation (6), the k index runs over all confined dimensions (d_{conf}), and the j index runs over the two opposite layers, both being adjacent to a confining hard wall. In the continuation, they will be referred to as "top" and "bottom" layers. In the case of bulk, unrestricted systems ($d_{\text{conf}} = 0$), the total crystallinity coincides with the bulk one, which is calculated, for example, in Equation (8) from [51].

From the technical perspective, we employ a step of mesh discretization of 0.1 rad for the orientation of the symmetry axis/axes. As mentioned earlier, the critical threshold for the detection of X-crystal similarity is set at $\varepsilon^{\text{thres}} = 0.245$. Unidimensional confinement, as studied here, corresponds to $d_{\text{conf}} = 1$ in Equation (6). Sphere monomers are assigned to the top or bottom layers for successive 2-D CCE analysis based on having an incomplete Voronoi polyhedron and very high corresponding value of the 3-D CCE norm. Practically, all atoms whose centers lie within a distance of $l_{\text{wall}} = (\sigma/2 + \sigma/4)$ from the closest wall belong to the top and bottom layers and are subjected to the 2-D CCE analysis independently of the system size ($n = 1200$ vs. 4800).

4. Results

Fully equilibrated system configurations at the end of the MC simulation for all systems studied here (N = 12, 24, 50 and 100) and at selected packing densities (φ = 0.50, 0.54, 0.58 and 0.61), with the coordinates of the sphere centers being subjected to periodic boundary conditions in the y and z dimensions, are shown in Figure 1. In these snapshots, the spheres are color-coded according to the parent chain.

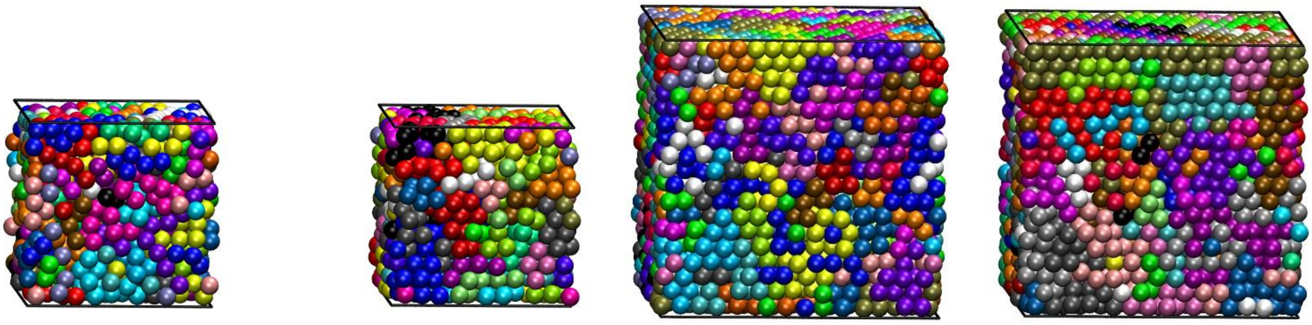

Figure 1. Snapshots of computer-generated configurations at the end of MC simulations. Left to right: 100 chains of N = 12 at φ = 0.50; 50 chains of N = 24 at φ = 0.54; 96 chains of N = 50 at φ = 0.58; and 48 chains of N = 100 and φ = 0.61. The sphere monomers are shown, with the coordinates of their centers being subjected to periodic boundary conditions in the y and z dimensions. The spheres are color-coded according to the parent chain. The flat hard walls along the confined (x) dimension are indicated by the black squares. The images were created with the VMD software [57]. The figure panels are also available in 3-D, interactive images.

The left panel of Figure 2 shows three randomly selected chains of the 48-chain N = 100 system at φ = 0.61, with the coordinates of the sphere centers fully unwrapped in the y and z dimensions. On the right panel, the full system is depicted as identifying the atoms that belong to the top and bottom layers of the system in contact with the hard walls, shown in yellow. All other atoms, shown in semi-transparent green, belong to the bulk volume of the system. Based on the discussion of the CCE structural descriptor in Section 3, in the continuation, we split the analysis of heterogeneous phase transition into surface and bulk crystallization. Accordingly, we apply the 2-D and 3-D CCE norms to calculate the order parameters for each individual reference crystal and thus the surface and bulk crystallinities. The total crystallinity is then readily available through the summation of the two contributions according to Equation (6).

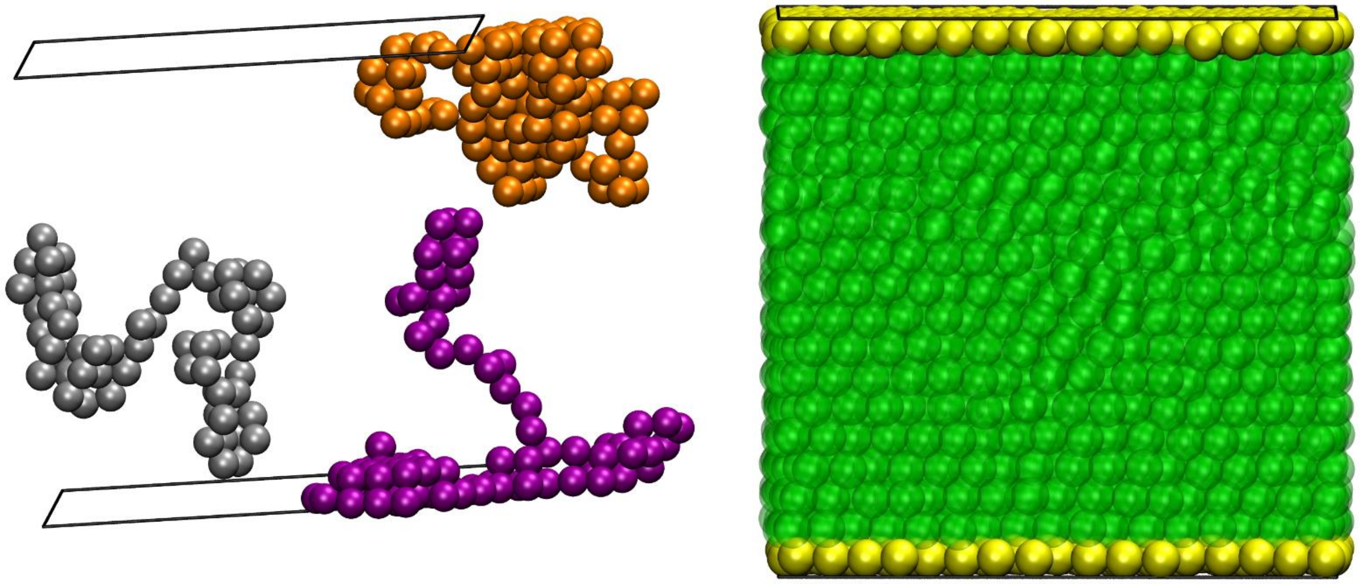

Figure 2. (**Left panel**): Snapshot showing three randomly selected chains of the 48-chain $N = 100$ system at $\varphi = 0.61$, with the coordinates of the sphere monomers fully unwrapped in the y and z dimensions. (**Right panel**): Layer identification for the CCE analysis. The atoms shown in yellow belong to the top and bottom layers adjacent to the walls, as they lie at a distance shorter than ($\sigma/2 + \sigma/4$) from the nearest wall. The green atoms, shown semi-transparently, belong to the bulk volume of the system. Accordingly, the yellow and green atoms are analyzed with the 2-D and 3-D CCE norm, respectively. The black squares identify the flat, parallel hard walls in the x dimension.

4.1. Total Crystallinity

For a given system of a fixed average chain length ($N = 100$) Figure 3 shows the evolution of the bulk (left panel) and surface (right panel) crystallinity as a function of the MC steps at all packing densities. First, with respect to the bulk behavior, far from the walls, systems of low volume fractions ($\varphi = 0.50$ and 0.52) show no traces of crystallites. We note here that for chains of tangent hard spheres in the bulk, the phase transition occurs at approximately $\varphi^{pol,bulk} \approx 0.58$, which is quite higher than the corresponding one of monomeric analogs ($\varphi^{mon} = 0.545$). Obviously, this threshold is not reached for the $\varphi = 0.50$ and 0.52 systems, even when the reduced packing density is considered ($\varphi_{wall} = 0.53$ and 0.55), incorporating the excluded volume effect of the impenetrable walls (Equation (4)). First, stable crystals are formed both in the bulk and on the surfaces once a density of $\varphi = 0.54$ ($\varphi_{wall} = 0.574$) is grasped. We observe here that by accounting for the wall effect the threshold of the reduced packing density where crystallization occurs is commensurate to the one of the unconstrained system. Beyond this point, there is a systematic increase of crystallinity with the increasing concentration. The maximum is reached for both τ^{bulk} and τ^{surf} at $\varphi = 0.60$. The next and final simulated volume fraction ($\varphi = 0.61$) shows similar trends, but the crystal nucleation and growth are slower, while the system reaches a less perfect final crystal morphology, judging from the absolute values of crystallinity.

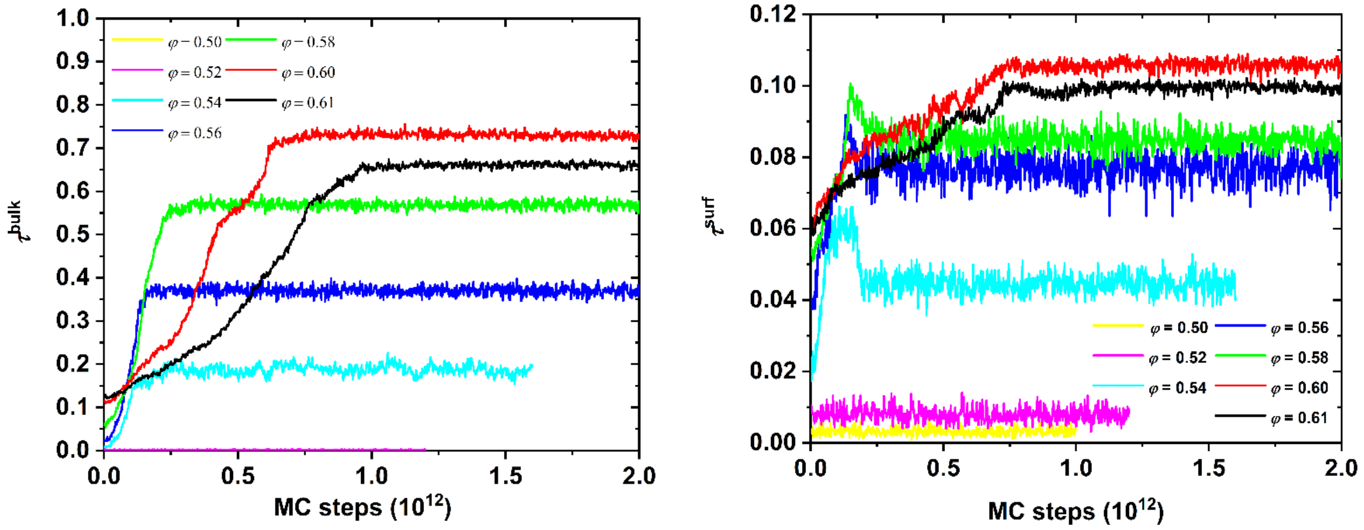

Figure 3. (**Left panel**) Bulk crystallinity, τ^{bulk}, and (**right panel**) surface crystallinity, τ^{surf}, as a function of the MC steps for the $N = 100$ system at various packing densities, φ.

It should be noted here that the surface crystallinity is calculated with respect to the whole set of atoms present in the system, the aim being that the total crystallinity, as calculated from Equation (6), adopts values in the interval [0,1]. Given that only a small fraction of atoms belongs to the top and bottom layers adjacent to the walls, this explains the low τ^{surf}, which does not exceed a maximum of approximately 0.11. As will be demonstrated in the continuation, only once the atoms that belong to the top and bottom layers are considered can the local surface crystallinity reach a unity corresponding to the crystal perfection of the surface. Comparing the trends of the two panels in Figure 3 the absolute increase is significantly higher for the bulk contribution, compared to the surface one. This can be explained, because there exist well-formed crystallites on the surface layers in the

initial configuration, which are migrated from less dilute systems during the phase of compression. By comparing the initial and final system configurations, the bulk crystallinity can increase by a factor of 7, while the surface one doubles at most.

With respect to the rate of crystallization Figure 4 shows the evolution of the bulk, surface, and total crystallinity as a function of the MC steps for the N = 100 system at volume fractions of φ = 0.60 and 0.61. It is apparent that the surface mechanism can be as slow as the bulk one when such high concentrations are reached. Even if there is a high initial fraction of crystallites on the wall surface, the densely packed environment of the top and bottom layers effectively hinders the significant re-arrangement of the local structure delaying perfection. From the trends of heterogeneous crystallization of athermal chains under unidimensional confinement, as established in Figures 3 and 4, it is evident that the surface crystallization has an important contribution in the total process and thus should be gauged with a high precision. Furthermore, the rate of the surface mechanism appears to be as slow as the one in the bulk volume of the system.

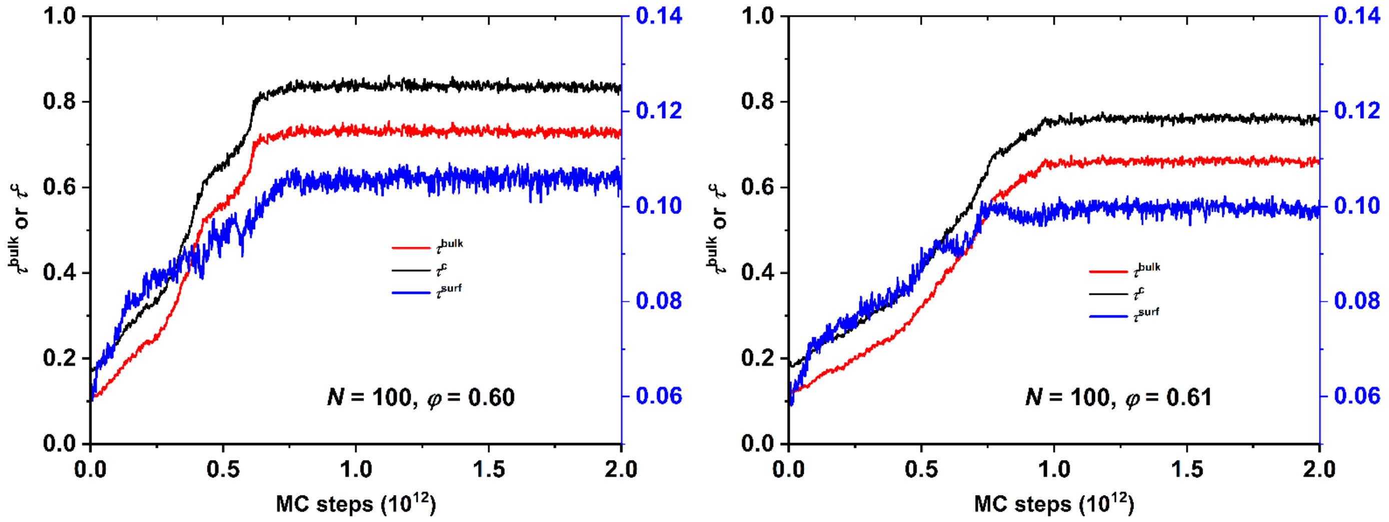

Figure 4. Double-y vs. x plots of bulk and total (left y-axis) and surface (right y-axis) versus the MC steps for the N = 100 system at packing densities of (**left panel**) φ = 0.60 and (**right panel**) 0.61.

Figure 5 presents, in linear-log (main) and linear-linear (inset) plots, the dependence of bulk, surface, and total crystallinities on the volume fraction for all the systems simulated here. Both the surface and bulk contributions increase with the packing density, with the surface being dominant at low packing densities (φ = 0.50 and 0.52), where only small isolated crystallites exist, and they are all dispersed on the wall surfaces. In contrast to unconstraint systems, where the phase transition is abrupt and reminiscent of a critical percolation phenomenon (compare, for example, Figure 5 with Figure 2 in [20]), the intermediate volume fractions here show a semi-crystalline system. Given the high heterogeneity of the confined system, especially with respect to the distribution of the sphere population, it is expected that the regimes near the wall are significantly more crystallized than the ones near the center of the cell and the farthest from the confining walls. Such information cannot be precisely identified from the trends shown in Figure 5 as the calculation of crystallinity entails the whole simulation cell. However, it is clear that the influence of the surface mechanism is dominant for low and intermediate volume fractions. At very high densities, most of the system has crystallized, as quantified by the very large fraction of sites with an ordered local environment. The highest value of total crystallinity is observed for the N = 50 system at φ = 0.61, where approximately 85% of the sites have either an HCP- or FCC-like similarity.

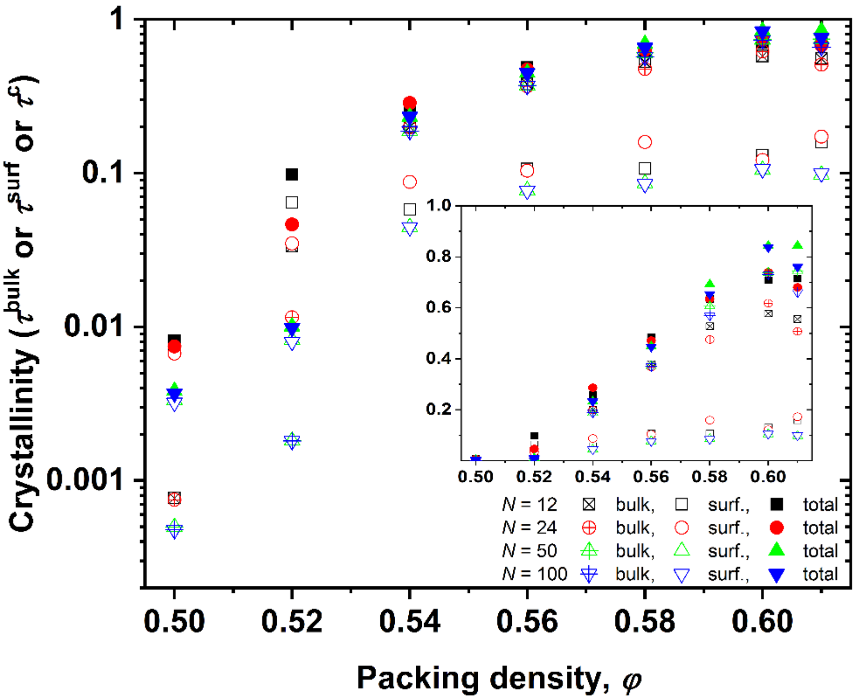

Figure 5. Linear-log plot of the dependence of bulk (τ^{bulk}), surface (τ^{surf}), and total (τ^{c}) crystallinity on the packing density, φ, for all simulated systems. Inset: same but in a linear-linear plot.

4.2. Local Density and Crystallinity

The highly heterogeneous nature of the simulated system due to the presence of the hard walls requires a further analysis based on the distance from (or proximity to) these confining agents. Therefore, we divide the simulation cell into layers with a width of 0.065 and calculate the corresponding profiles of the local density and CCE-norm order parameter as a function of the position along the dimension of confinement. Such profiles can further include the evolution of the system in (simulation) time, measured in Monte Carlo steps. In the continuation coordinates along the x-dimension are rescaled so that the extreme values of −1 and +1 correspond to the position of the bottom and top hard walls, respectively, while 0 designates the center of the simulation cell.

Figure 6 hosts stacked plots of the local density profiles as a function of the coordinates for the $N = 100$ system, as obtained from the final and stable phase of the production MC simulation. Different plots correspond to different packing densities, with the coloring scheme following that shown in Figure 3. As the concentration increases, the system becomes progressively more homogeneous. At the lowest concentration (φ = 0.50), after the depletion layer due to excluded volume from the hard wall, a sharp peak in local density is observed, with the two following maxima indicating the formation of local layers. For longer distances, the distribution becomes uniform, with an average value significantly smaller than the one of the top and bottom surface layers. Increasing the volume fraction to φ = 0.52 slightly prolongs the population and intensity of these high-concentration layers near the walls. At φ = 0.54, the system shows signs of layer morphologies but only in one (top) side, as the other remains in the same state as in lower volume fractions. The important difference is triggered once φ = 0.56 is reached. Characteristic density layers extend and cover the totality of the simulation cell. As the distance increases from the confining walls, the intensity of the concentration maxima decreases, a trend particularly evident in the central region of the cell. As the concentration further increases, the density profile becomes even more uniform along the confined dimension. Still, the sphere population on the walls is significantly higher than that away from them. At the highest density studied (φ = 0.61), the density profile is less uniform than at φ = 0.60, a trend consistent with the lower crystallinity, as demonstrated in Figure 5. This is due to

the crowded environment that prohibits an efficient rearrangement of the local structure. The situation does not change even if the MC simulation is significantly extended. This present simulation finding is in agreement with the pioneering experiments of Pusey and van Megen [45], according to which a very high concentration hinders crystallization so that the expected, thermodynamically stable crystals are not formed or correspond to a defect-ridden and incomplete structure, even when the experimental observation time is prolonged.

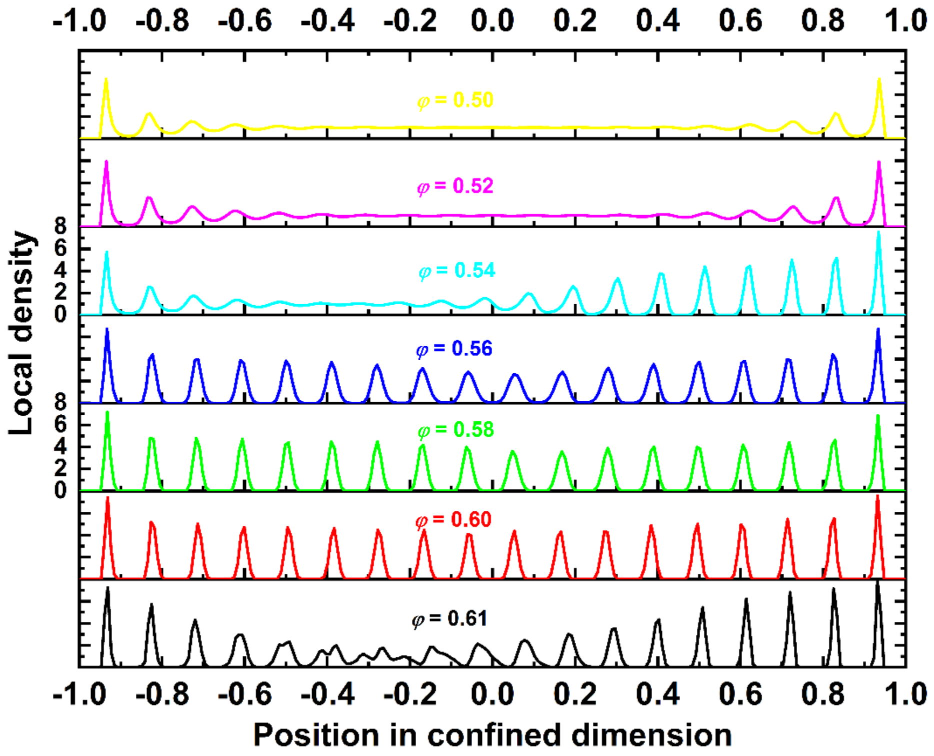

Figure 6. Stacked plots of local density versus the position in the confined (x) dimension for the N = 100 system. −1, 0, and +1 correspond to the positions of the bottom wall, center of the simulation cell, and top wall, respectively. Different plots correspond to different packing densities, φ. The coloring scheme follows that shown in Figure 3.

The evolution of the local density profile as the MC simulation progresses is presented in Figure 7 for the N = 100 system at φ = 0.60. It is evident that as the simulation advances, the layered morphology of well-shaped regimes of low and high concentrations, initially evident only near the walls, propagates and covers the total volume of the simulation cell. Additionally, near the end of the simulation, all density layers in the bulk of the system adopt regular positions of very comparable peak intensities, indicative of crystal ordering. The extreme top and bottom layers still have a higher concentration of particles, but the density profile along the confined dimension for the highest volume fractions ($\varphi \geq 0.56$) is significantly more uniform than in the case of predominantly amorphous (φ = 0.50 and 0.52) or semi-crystalline (φ = 0.54) structures.

Analogous plots, like those presented above for density, are presented for local crystallinity. The local order parameter with respect to a specific X crystal is now defined as the fraction of sites with X similarity, as gauged by the CCE-norm analysis, divided by the total number of sites inside the volume of the sub-domain (layer of thickness 0.065) along the confined dimension. Figure 8 shows stacked plots of local crystallinity as a function of the position. Different plots correspond to different packing densities. The lines and symbols correspond to the bulk volume (3-D CCE) and surface layers (projected 2-D CCE), respectively. The trend of the local crystallinity very closely follows that of the local

density with respect to the effect of the volume fraction. Along the bulk layers, no crystallites exist for the φ = 0.50 system and very few and randomly dispersed ones appear at φ = 0.52. In both cases, surface layers are characterized by a higher local degree of ordering than the bulk ones. At φ = 0.54, crystallization occurs, but it covers only one part (top) of the simulation cell. Interestingly, even the projected 2-D crystallinity on the bottom layer remains at very low levels. At higher densities ($\varphi \geq$ 0.56), the crystallization becomes more homogenous: surface layers have a high degree of ordering, which extends further to bulk ones. At φ = 0.56 and 0.58, only a small fraction at the center of the simulation cell, the farthest possible distance from the confining agents, remains amorphous. At an even higher density (φ = 0.60), the local order parameter is almost a unity on the surface, indicative of crystal perfection. Bulk layers are also characterized by a very high degree of ordering of very similar intensities. In all cases, the trends of the crystallization profile (Figure 8) are very reminiscent of the ones for density (Figure 6). Therefore, the evolution of local density along the dimension of confinement can be used as a preliminary measure of the phase transition of hard-sphere chains under flat confinement.

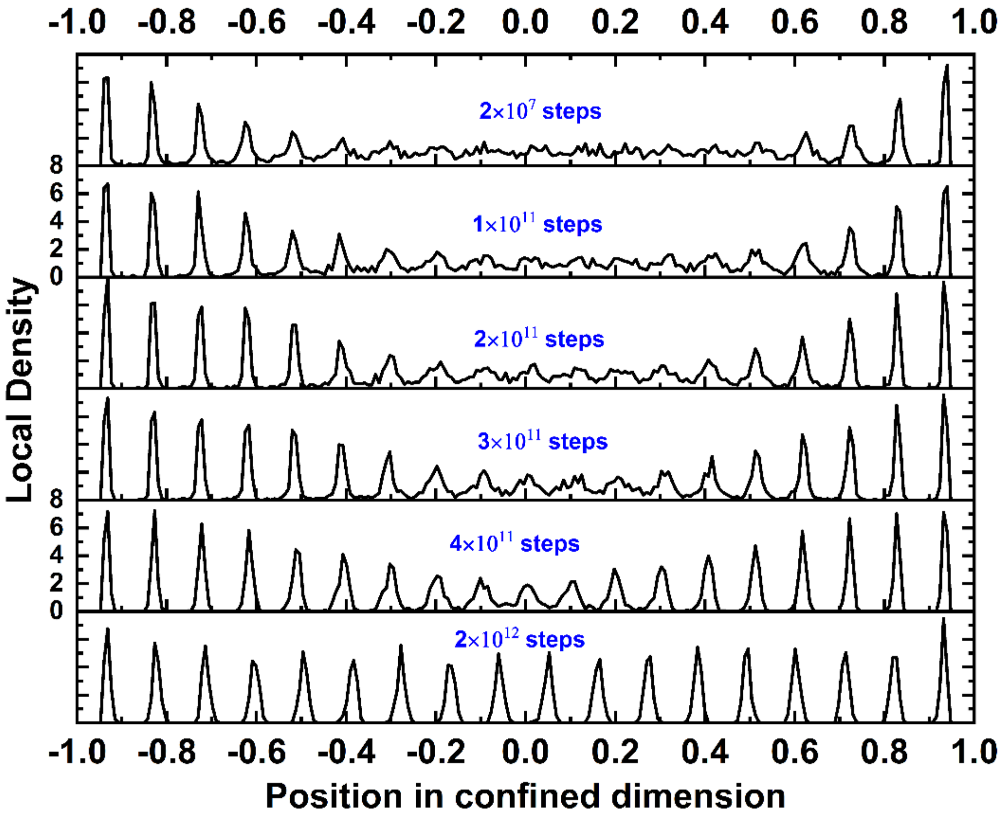

Figure 7. Stacked plots of the local density profile as a function of the position in the confined dimension for the N = 100 and φ = 0.60 system. Different plots correspond to different steps (frames) along the MC trajectory.

Another interesting aspect of high-density crystallization, of polymers [14,15,20,58,59], or monomers [55,56] is the competition between the formation of local structures with a fivefold symmetry and the nucleation and growth of crystallites of an HCP/FCC character. Figure 9 shows the local order parameter for the HCP/FCC crystals (left panel) and the FIV symmetry (right panel) as a function of the position along the dimension of confinement for the N = 100 system. The curves have been obtained by sampling over different configurations in the equilibrated part of the MC trajectories, and different plots correspond to different packing densities. Blue, red, and green correspond to the HCP, FCC, and FIV order parameters, respectively.

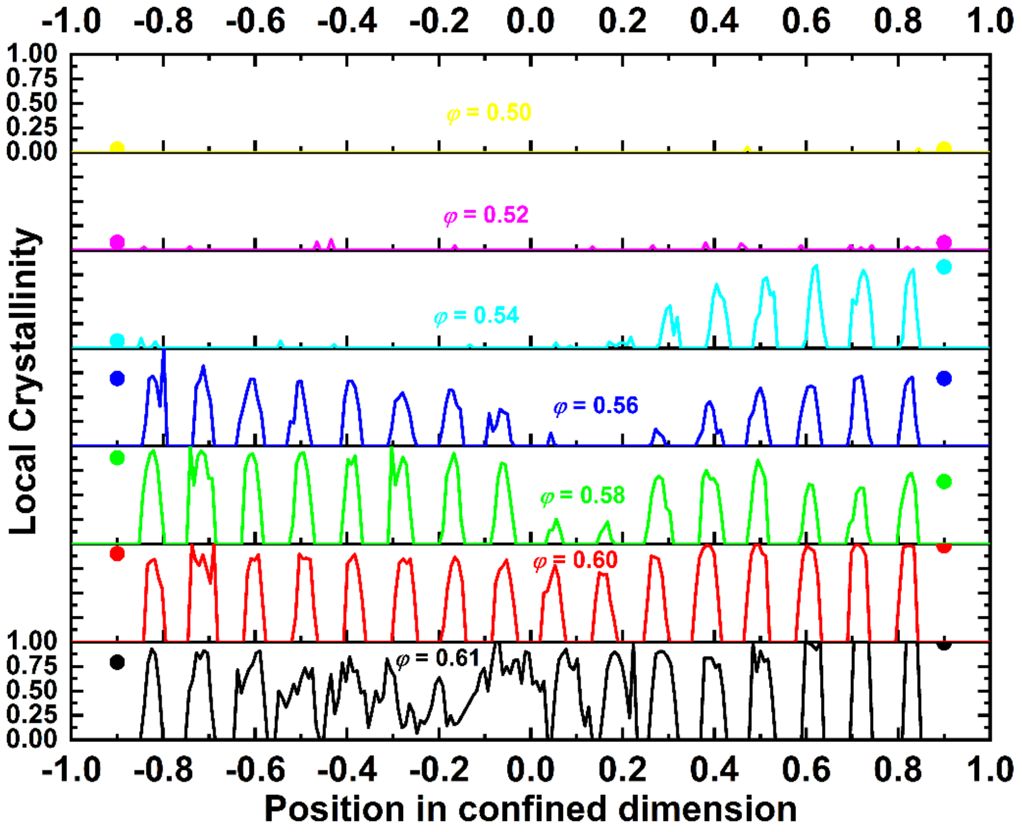

Figure 8. Stacked plots of the local crystallinity, as quantified by the CCE norm as a function of the position in the confined dimension for the $N = 100$ system. Different plots correspond to different packing densities. The lines and symbols correspond to the bulk and surface local crystallinities, respectively.

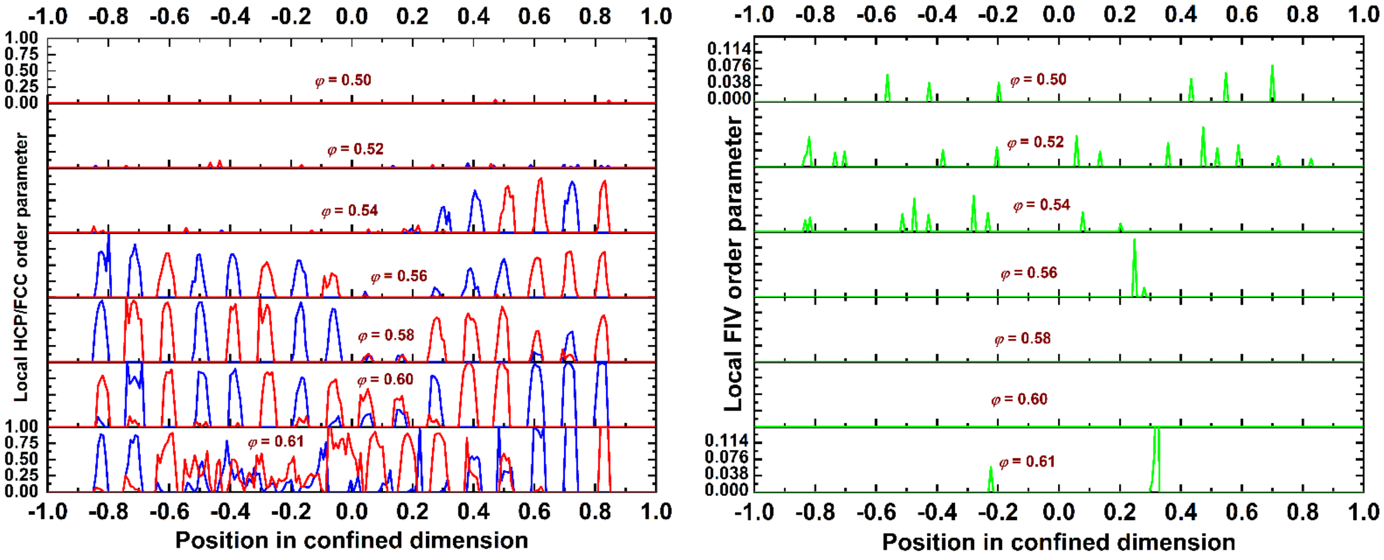

Figure 9. Stacked plots of the local (**left panel**) HCP/FCC and (**right panel**) FIV order parameter, as calculated from the CCE norm, as a function of position in the confined dimension for the $N = 100$ system. Different plots correspond to different packing densities. Blue, red, and green correspond to the HCP, FCC, and FIV order parameters, respectively.

The curves shown in Figure 9 allow for a better understanding of the established crystal morphologies or of their absence. At the lowest simulated density, there are no traces of HCP or FCC crystallites, while there exist fivefold-like sites, which are dispersed along the volume of the simulation cell. As the density increases, so does the population

of spheres with a fivefold symmetry, which is similar to what happens in bulk (unconstraint) packings of monomers [55]. At φ = 0.52, the sites of an FIV character appear to be randomly scattered along the confined dimension. However, at even higher concentrations, the structural competition between fivefold formation and crystal growth settles in. For example, at φ = 0.54, FIV sites dominate in the amorphous (bottom) half of the system, while they are completely absent from the ordered (top) half, which is populated by alternating layers of a pure HCP or FCC character. As the density increases to φ = 0.56, the system becomes highly ordered, with the crystal growing and migrating from near the walls to the inner volume of the cell. In parallel, FIV sites are forced to exist in the remaining amorphous regions of a low crystallinity. As the volume fraction further increases (φ = 0.58 and 0.60), the system becomes uniformly ordered, and the FIV sites disappear, as their presence—and twinning in general—is incompatible with the layered morphology of athermal polymer crystals [15]. At an even higher concentration (φ = 0.61), as crystallization becomes less perfect, the FIV sites reappear in regions of a low crystal order. If we move our attention to the ordered layers (left panel of Figure 9), the majority of these have a pure HCP or FCC character, and in many instances, they are free of impurities in the form of amorphous (AMO) sites. Less frequent, but still possible, is the occurrence of close-packed layers of a mixed HCP/FCC character. In all cases, layers are formed with a single stacking direction parallel to the dimension of confinement.

In the continuation, we study how these ordered layers are initially formed and then grow along the simulation cell. This information is available through the stacked plots shown in Figure 10. The left and right panels show the evolution of the HCP/FCC and FIV local order parameter profiles, respectively, as the MC simulation progresses for the N = 100, φ = 0.60 system. A side-by-side comparison unmistakably reveals the structural competition between crystallization and fivefold formation. Initially, the crystals nucleate and grow near the confining walls. In parallel, the FIV sites exist in significant numbers in the inner parts of the cell, far from the top and bottom surfaces. As the MC simulation advances, the crystals show perfection at and near the walls, with the formation of layers of a mixed HCP and FCC character. In parallel, the FIV sites are completely eliminated from these regimes. The more the crystal layers expand from the surfaces to the center of the cell, the smaller the amount of volume available for fivefold formation. The fivefold population is progressively ostracized to the central part of the cell. Eventually, when this regime is further occupied by HCP and FCC crystals, the FIV sites are eliminated. In all studied systems, the presence of hard walls always leads to the formation and growth of alternating HCP/FCC layers with a unique stacking direction. In the past, we have demonstrated, through simulations and theoretical arguments, that such a chain crystal morphology is not compatible with twinning and, as such, with fivefold formation in the form of structural defects [15]. Focusing on the surface mechanism, very few or no pentagonal sites are detected on the hard walls.

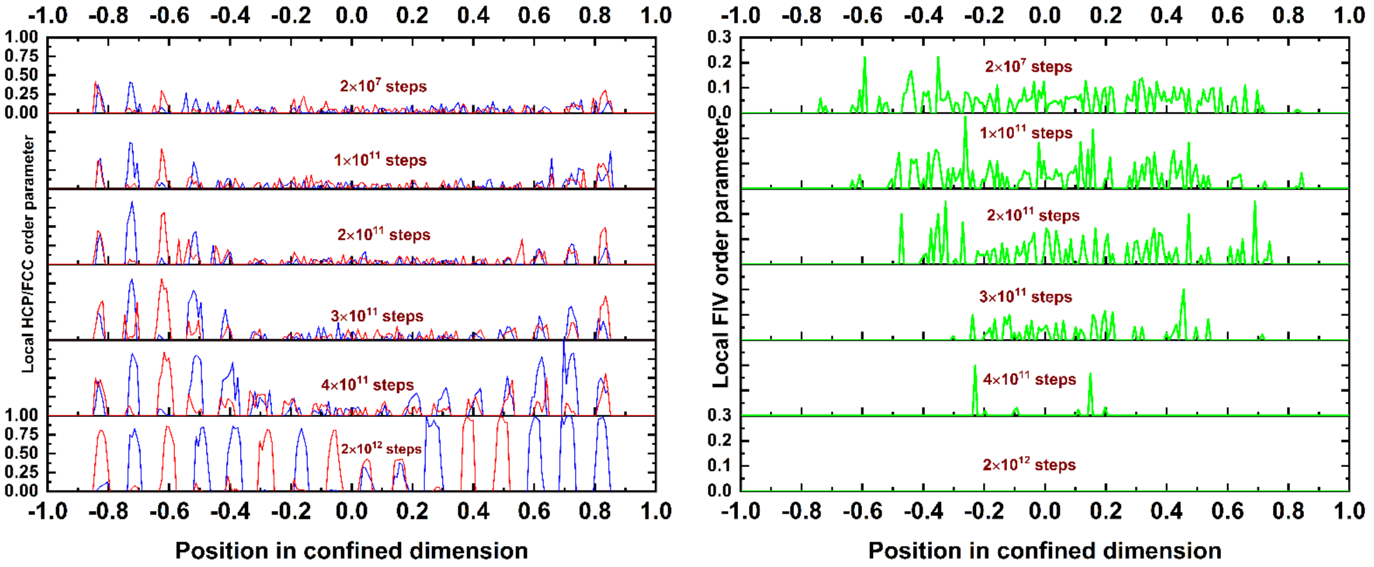

Figure 10. Stacked plots of local (**left panel**) HCP/FCC and (**right panel**) the FIV order parameter, as calculated from the CCE norm, as a function of the position in the confined dimension for the N = 100 system at φ = 0.60. Different plots correspond to different MC steps. Blue, red, and green correspond to the HCP, FCC, and FIV order parameters, respectively.

4.3. Snapshots of Computer-Generated Configurations

Structural analysis through the local order parameter, as quantified through the CCE norm, has provided an accurate identification of the established crystal morphologies, especially for the bulk mechanism. Next, we provide additional evidence of crystal nucleation and growth through visualization of the computer-generated system configurations, especially with respect to the surface mechanism. In all system snapshots to be presented in the continuation, the following coloring convention is used, according to the CCE-based analysis: HCP (blue), FCC (red), FIV (green), TRI (cyan), SQU (purple), HON (orange), and PEN (green). Amorphous or unidentified sites (AMO) are shown in yellow and with reduced dimensions (in bulk snapshots) for visual clarity. Due to the high heterogeneity of the confined polymer system, two separate snapshots are required to visualize the top and bottom layers (surface crystallization), as well as one for the inner volume (bulk crystallization) of the simulation cell. The hard walls and borders of the simulation cells are indicated by the thick black squares.

Figure 11 presents snapshots of the final configurations for the N = 100 system at φ = 0.50, 0.54, 0.56, and 0.60. The top and bottom panels correspond to the top and bottom surface layers, which are identified through the projected 2-D CCE norm, while the middle panel corresponds to the whole system and is gauged by the 3-D CCE norm. All atoms belonging to the top and bottom layers of the middle panels in Figure 11 apear as amorphous or, more precisely, as "unidentified". As explained in Section 3 this is because the 3-D CCE norm cannot detect order in these atoms, which is expected, because they cannot create 3-D crystals due to the presence of the flat surfaces. One must therefore resort to the projected 2-D CCE norm for a quantitative description of the surface layers (as seen in the top and bottom panels of the same figure).

Visualization of the computer-generated polymer morphologies further validates the trends established from the profiles of the CCE-based local order parameters shown in Figures 8 and 9. At φ = 0.50, the bulk volume is amorphous, except for very few isolated sites with an ordered or fivefold similarity. Likewise, the top and bottom layers have comparatively low surface coverages and very few sites as crystallites. At low densities, these sites can possess a TRI or SQU local environment. At φ = 0.54, a clearly highly heterogeneous semi-crystalline structure is established, as reflected in both the surface and the bulk panels. A further increase in density increases the number of ordered layers, and the

amorphous and fivefold sites exist only in the central, low-density regimes of the simulation cell. At φ = 0.60, HCP and FCC layers cover the bulk of the system, while the top and bottom surfaces are covered by sites with a TRI character. Isolated amorphous sites exist both in bulk and on the surface as structural defects. Most of the bulk crystal layers have a unique character: pure HCP or FCC similarity, especially if they lie near the hard walls. Layers of a mixed HCP/FCC type are observed primarily in the central part of the simulation cell.

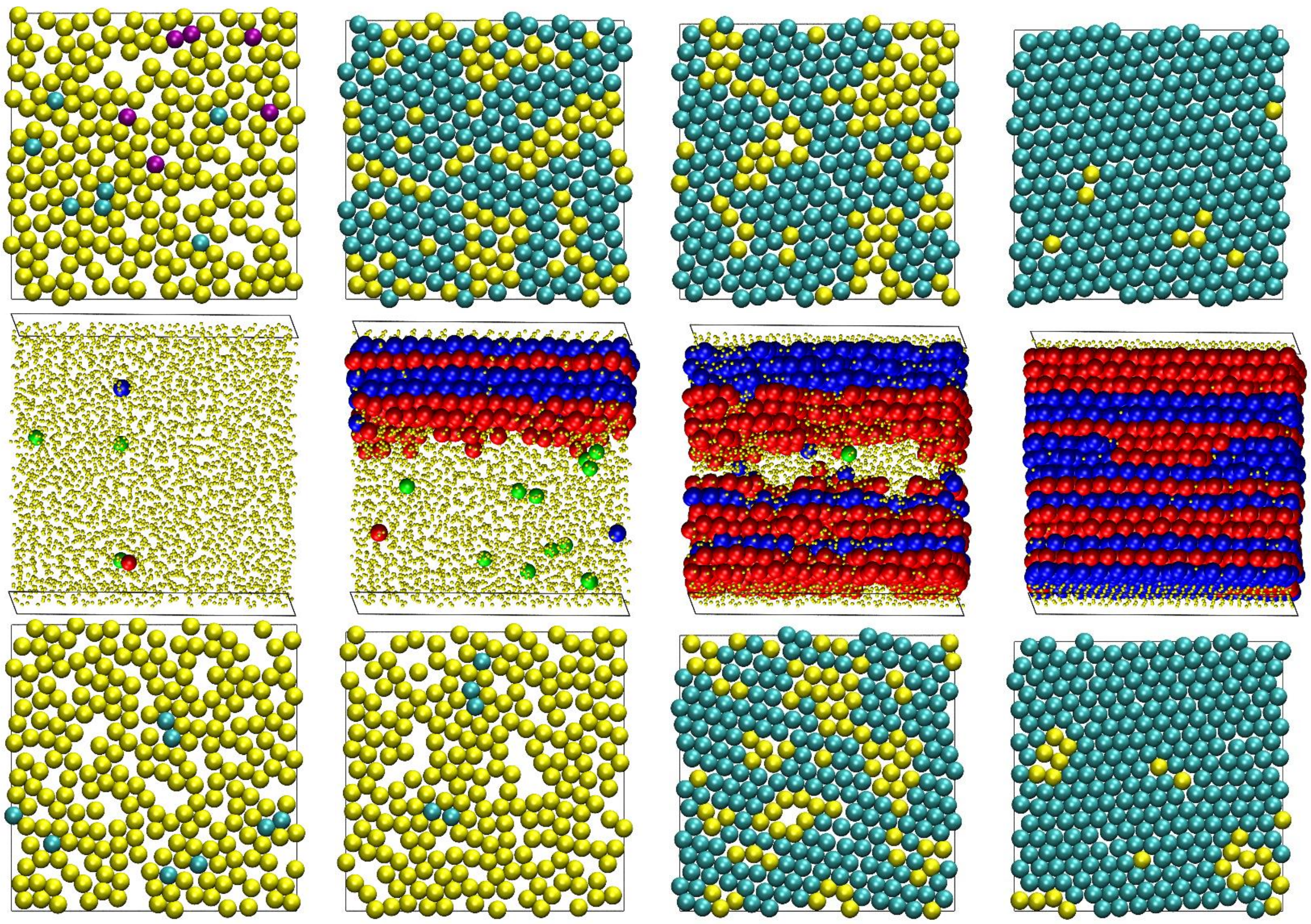

Figure 11. Snapshots of the system configurations for the N = 100 at φ = (from **left** to **right**) 0.50, 0.54, 0.56, and 0.60 at the end of the MC simulation. The (**top**), (**middle**), and (**bottom**) panels correspond to the top layer, bulk volume, and bottom layer of the confined simulation cell. The atoms are colored-coded according to the crystal similarity, as identified by the CCE-norm analysis. For bulk crystallization (3-D CCE), blue, red, and green correspond to the HCP-, FCC-, and FIV-like sites, respectively. For surface crystallization (2-D CCE), cyan, purple, orange, and green correspond to the TRI-, SQU-, HON-, and PEN-like sites, respectively. The amorphous (AMO) sites are colored in yellow. In 3-D, the AMO sites are further shown with reduced dimensions (in a 1:5 ratio) for visual clarity. The figure panels are also available as 3-D, interactive images.

In the snapshots shown in Figure 11 triangular symmetry appears to be the predominant morphology for surface crystallization at high volume fractions. This is expected, as among all possible 2-D crystals, the triangular one corresponds to the highest surface coverage. However, it is not the only ordered structure that is encountered in the present simulations as seen in Figure 12 at the end of the MC simulations for selected N = 12 and 24 systems. Crystal perfection is apparent for the N = 24 and φ = 0.58, where the projected surface morphology corresponds to a single triangular crystal. Such triangular morphol-

ogies, which correspond to the densest packings in 2-D, are in agreement with the experimental realizations of monomeric hard spheres in extreme, single-layer confinement, as reported in studies of colloidal analogs [46]. Still, SQU-dominated structures are also generated, as well as ones of a mixed SQU/TRI type, with amorphous atoms existing as defects at the boundaries of the two different crystal morphologies.

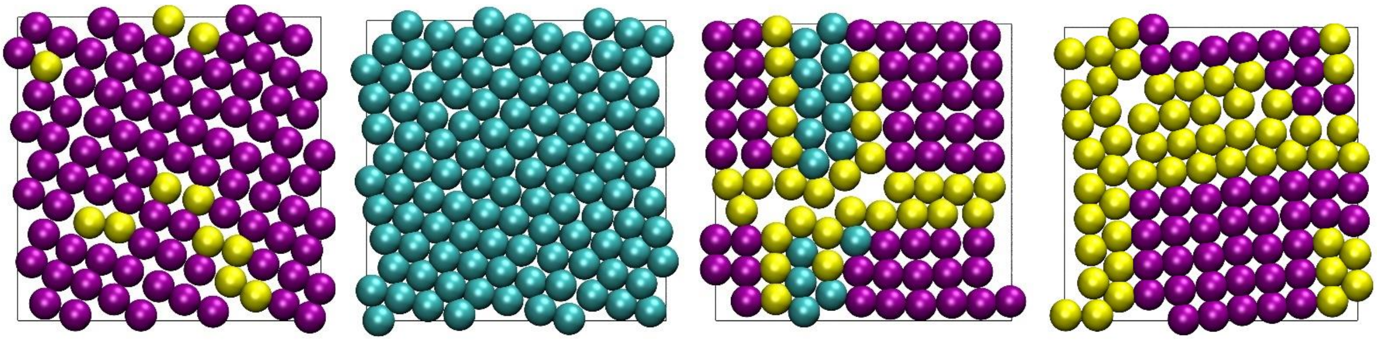

Figure 12. Projected surface morphologies at the end of the MC simulations, as identified by the 2-D CCE-norm analysis for the (from **left** to **right**): $N = 12$, $\varphi = 0.56$ (top layer); $N = 24$, $\varphi = 0.58$ (bottom layer); $N = 12$, $\varphi = 0.60$ (top layer); and $N = 12$, $\varphi = 0.61$ (top layer) systems. The figure panels are also available as 3-D, interactive images.

Figure 13 visually presents the evolution of the surface and bulk crystallinity at different instances of the MC simulation on the $N = 100$ and $\varphi = 0.60$ system. First, near and at the surfaces, the degree of ordering is significant. Almost half of the population of surface atoms have a crystal similarity, while the defect-ridden layers of an HCP/FCC character exist adjacent to the walls. This high level of ordering is caused by the compression from lower volume fractions. It also marks a significant difference with respect to the athermal chain crystallization of unrestricted (bulk) systems under the same conditions, where the initial packings are characterized by a very low degree of ordering. In the bulk volume far from the walls, fivefold-like sites exist in abundant numbers. As the simulation progresses, their numbers are reduced, they get "exiled" to the central parts of the cell, and they eventually disappear as the whole simulation cell is covered by alternating HCP and FCC layers with a unique stacking direction, parallel to the dimension of confinement. The projected 2-D crystallization follows similar patterns, except for the fact that it starts from a comparatively higher fraction of ordered sites. In this specific example, SQU-like sites exist initially in the bottom layer. However, the SQU population is gradually reduced, and both surfaces are converted into 2-D structures of a triangular morphology. While the surface crystallinity is initially high, the corresponding mechanism leading to 2-D crystal perfection is not significantly faster than the one for the bulk volume.

In the Supplementary Material, a 3-panel video is available on the crystal nucleation and growth, as observed for both the surface and bulk mechanisms of hard-sphere chain crystallization under unidimensional confinement for the $N = 100$ system at $\varphi = 0.60$.

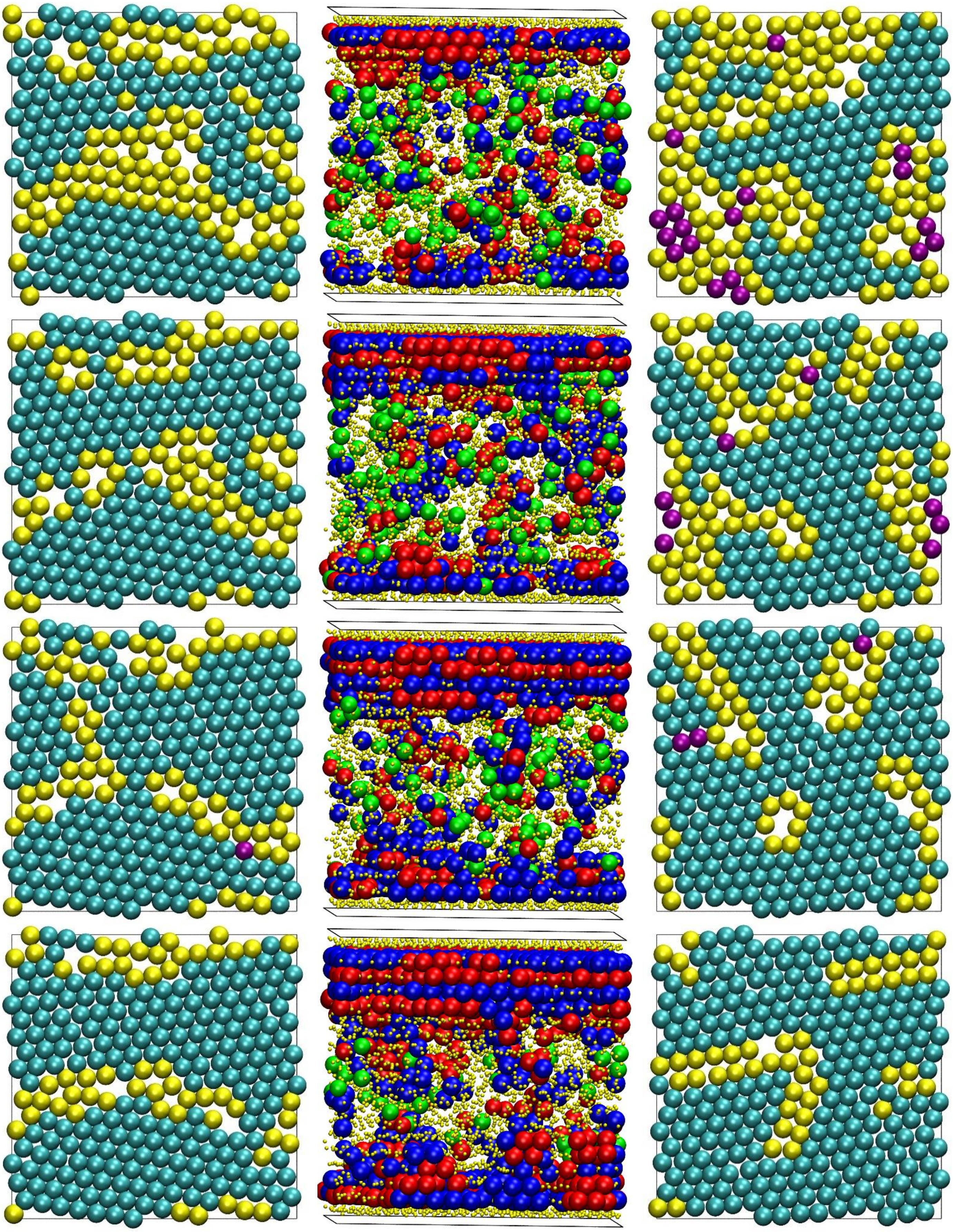

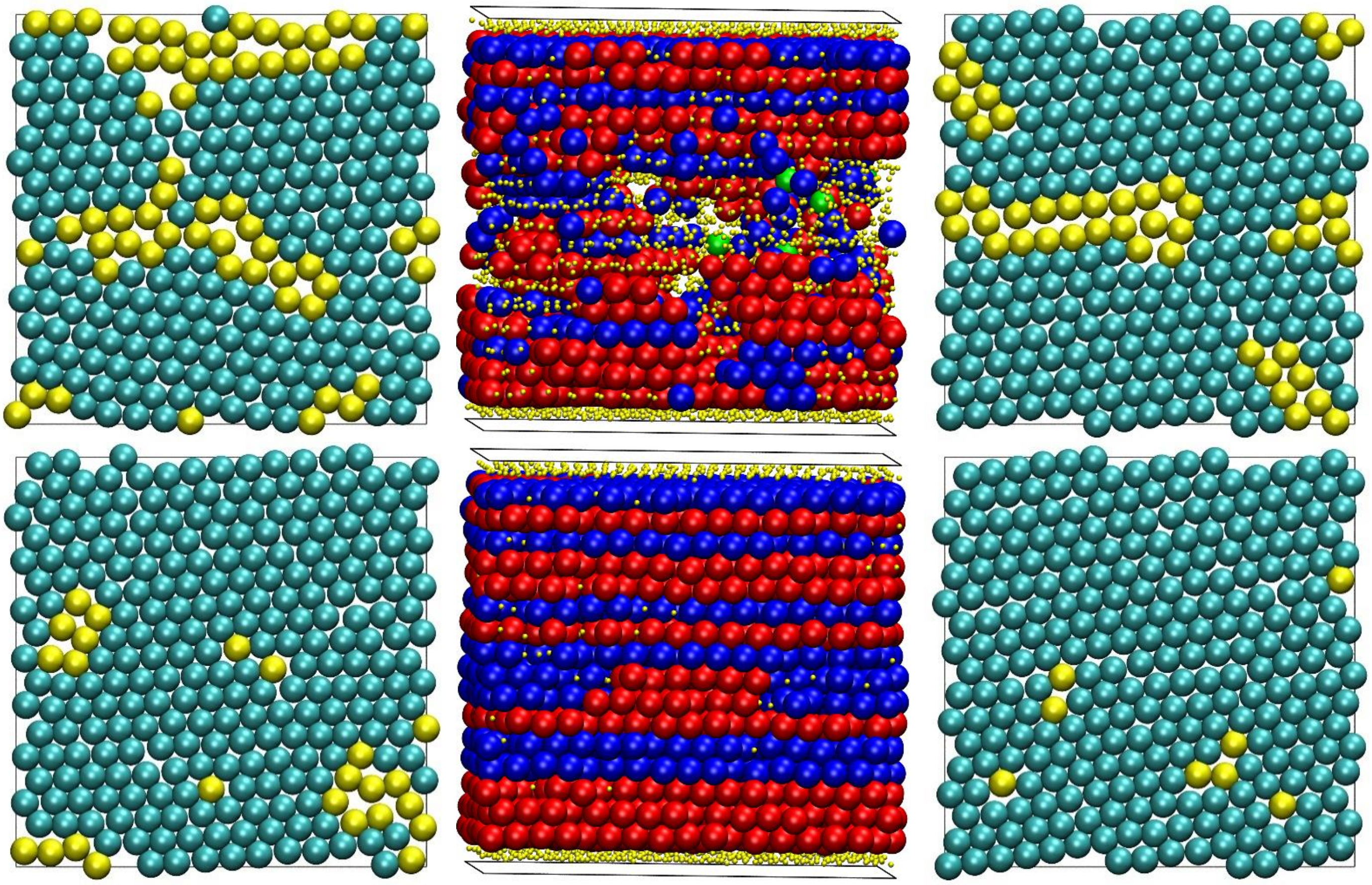

Figure 13. Snapshots of the system configurations for the $N = 100$ at $\varphi = 0.60$ at various instances along the MC simulation. (**Left**), (**middle**), and (**right**) panels correspond to the bottom layer, bulk volume, and top layer of the confined simulation cell. From (**top**) to (**bottom**): 2×10^7, 1×10^{11}, 2×10^{11}, 3×10^{11}, 4×10^{11}, and 2×10^{12} MC steps. The atoms are colored-coded according to the crystal similarity, as identified by the CCE-norm analysis. The figure panels are also available as 3-D, interactive images.

5. Discussion

As a final note, the results of the structural analysis, based on the CCE-norm descriptor, entail an uncertainty related to labeling a site as having an X-crystal similarity by adopting a threshold (which is here equal to 0.245 for both the bulk 3-D and projected 2-D mechanisms). It is to be understood that a site i with an X-CCE norm very close to zero ($\varepsilon_i^X \to 0$) is structurally more similar to the reference crystal X, compared to a site with a norm still lower but very close to the threshold ($\varepsilon_i^X \to \varepsilon^{\text{thres}}$). Both sites are identified as X-like. Altering this threshold to stricter values reduces the fraction of such sites, while increasing it has the opposite effect. The specific value is selected so as to minimize or eliminate the possibility of a site having a dual character, and it is a result of the observation of all possible parity plots of the reference crystals, as explained in detail in [50,51]. A computer-generated crystal environment, especially when produced from an initial random packing, is inescapably ridden by structural imperfections, compared to the ideal reference crystal. Examples of local environments with progressively reduced similarities to both 2-D and 3-D references are given in [51].

All qualitative trends presented here on crystal nucleation and growth, either on the surface or in the bulk of the system, will not change if the corresponding CCE thresholds become softer or harder. This has also been established in previous publications on athermal or energetically-driven systems analyzed through the CCE-norm descriptor [16,20,21,55,56,58,59].

With respect to the Monte Carlo simulation, we should mention that at high concentrations (packing densities), like the ones studied here, ergodicity is not guaranteed. Sampling of configurational space is achieved by the combination of the MC algorithms, including the chain-connectivity-altering ones. The latter, as implemented here, do not involve sphere displacement(s), so the high packing density does not hinder their performance. In fact, due to the increased number of contact neighbors, as the volume fraction increases, their performance improves, especially near jamming [49]. By having high acceptance rates, chain-connectivity-altering moves provide a different chain contour and connectivity, offering new paths in the configuration space for the local moves to occur. Thus, a synergy is set between the different types of MC moves. Still, at very high densities, the sampling of configurational space can be problematic. This is the reason why simulations are extended to cover 10^{12} MC steps. Furthermore, based on the presented results, it is clear that at $\varphi = 0.61$, the obtained crystals are not as perfect as those obtained at a lower concentration ($\varphi = 0.60$), and such a trend of structural imperfection with increasing density prevails as we approach jamming. In parallel, this tendency has an analogy with the findings of colloidal experiments, where once a packing density is exceeded, glass formation, often denoted as "Bernal glass" [45], is observed, while the close-packed crystal is the thermodynamically stable and thus the expected structure.

In parallel, we need to state again that the present MC simulations should not be interpreted dynamically, as such information is not available due to the stochastic nature of the method and the application of the unphysical, but highly-efficient, chain-connectivity-altering algorithms.

The employed model of freely-jointed chains of tangent hard spheres of a uniform size shows a phase behavior reminiscent of that of monomeric counterparts. As such, ordered morphologies consist of stackings of close-packed crystallites of sites with a face-centered cubic (FCC) and/or hexagonal close-packed (HCP) character. In parallel, at the level of chains, these show no stretching or orientational order, as observed in the crystallization of "traditional" polymers, for example, in polyethylene. The model adopted here to describe polymer chains, as it lacks chemical detail, deviates from the ones of chemical complexity used to study atomistic or coarse-grained systems, such as the ones in the early works of [60,61]. Our simulation findings and trends should be compared with the ones of colloidal or granular chains, especially when the colloidal suspensions are synthesized to have a hard-sphere-like behavior.

Finally, given the athermal nature of the hard sphere model, entropy is the sole driving force behind the phase transition. As has been demonstrated in our past simulations of athermal chains in the bulk, crystallization occurs because the local environment around each site becomes more spherical and more symmetric, as order is introduced at the local level [14,16,20]. This, in turn, increases the translational entropy of the system, which more than compensates for any loss in the configurational parts. Spheres can move with more freedom in a local environment whose symmetry increases its available volume. At the global level, freely-jointed chains in the crystal behave as random walks, while the corresponding close-packed crystals are structurally quite perfect, as demonstrated here.

6. Conclusions

We present a description of the mechanism of the crystal nucleation and growth of polymers of tangent hard spheres under unidimensional confinement, as observed in extensive MC simulations. Confinement is realized through the presence of impenetrable and parallel hard walls. We analyze crystallization through two different mechanisms: surface and bulk crystallization, each quantified through the application of the CCE-norm descriptor. Atoms lying close to the confining walls are projected on the surface, as they have an incomplete local environment due to the proximity to the flat walls. The total degree of crystallinity and the final stable morphologies depend strongly on the packing density, while the chain length has no effect, as in the case of unconstraint chain systems.

With respect to bulk crystallinity, the resulting ordered crystals consist of random hexagonal closed-packed layers with a unique stacking direction perpendicular to the confining walls. Such layers are of a pure HCP of FCC constitution, except for the ones that are formed the latest and the farthest from the flat surfaces. The structural competition between bulk crystallization and fivefold formation is also evident here. The final layered bulk polymer crystals are free of twin defects. As far as the surface crystallization is concerned, the perfection mechanism is found not to be significantly faster than the bulk one, although the surface is initially covered by a higher number of crystal nuclei. This can be explained as the top and bottom layers, especially for high volume fractions, are characterized by significantly higher concentrations of atoms, compared to the volume of the cell. Thus, the structural rearrangements on the wall surfaces are frequently hindered by the crowded environment, compared to the more dilute volume of the system far from the hard walls. The final surface morphologies have a predominantly triangular character, but there exist cases where principal domains of square crystals or of a mixed TRI/SQU character are formed. In sharp contrast to the bulk mechanism, no appreciable population of sites with a pentagonal symmetry is detected on the surfaces, especially at the early stages of crystallization.

The present work has placed emphasis on the description of the global mechanism for the surface and bulk crystallization of athermal polymers. Future research will focus on explaining the entropic origins of the phenomenon and compare them with the unconstraint (bulk) case through a detailed analysis of the statistics of the Voronoi polyhedra and polygons and the ability of chain monomers to rattle (flip or rotate) near to and far from the surfaces.

Supplementary Materials: The following are available online at www.mdpi.com/xxx/s1: All figure panels in 3-D, interactive format. A 3-panel video showing the surface and bulk crystallization for the $N = 100$ system at $\varphi = 0.60$. A 3-D, interactive version of the present manuscript.

Author Contributions: Conceptualization, N.C.K.; methodology, K.F., N.C.K. and M.L.; software (MC), P.M.R., M.H., N.C.K. and M.L.; software (CCE) P.M.R., K.F., N.C.K., and M.L.; data curation, P.M.R., M.H., K.F., N.C.K. and M.L.; writing—original draft preparation, P.M.R. and N.C.K.; writing—review and editing, M.H., K.F. and M.L.; visualization, P.M.R., M.H. and N.C.K.; funding acquisition, K.F., N.C.K. and M.L. All authors have read and agreed to the published version of the manuscript.

Funding: This research was funded by MICINN/FEDER (Ministerio de Ciencia, Innovación y Universidades, Fondo Europeo de Desarrollo Regional), grant numbers "MAT2015-70478-P" and "RTI2018-097338-B-I00", and by UPM and Santander Bank, "Programa Propio UPM Santander".

Institutional Review Board Statement: Not applicable.

Informed Consent Statement: Not applicable.

Data Availability Statement: The presented simulation data are fully available upon request.

Acknowledgments: The authors acknowledge the support through the projects "MAT2015-70478-P" and "RTI2018-097338-B-I00" of MICINN/FEDER (Ministerio de Ciencia, Innovación y Universidades, Fondo Europeo de Desarrollo Regional). M.H. acknowledges the financial support through the "Programa Propio UPM Santander" of UPM and Santander Bank. The authors gratefully acknowledge the Universidad Politécnica de Madrid (www.upm.es) for providing the computing resources of the Magerit Supercomputer through the projects "p208", "r553", and "s341".

Conflicts of Interest: The authors declare no conflict of interest. The funders had no role in the design of the study; in the collection, analyses, or interpretation of data; in the writing of the manuscript, or in the decision to publish the results.

Abbreviations

The following abbreviations are used throughout the manuscript:

AMO	Amorphous
BCC	Body-centered Cubic
CCE	Characteristic Crystallographic Element (norm)
FCC	Face-centered Cubic
FIV	Fivefold
HCP	Hexagonal close-packed
HEX	Hexagonal
HON	Honeycomb
HS	Hard Sphere
MC	Monte Carlo
PBC	Periodic Boundary Condition
PEN	Pentagonal
SQU	Square
TRI	Triangular

References

1. Chen, Y.S.; Yao, Z.W.; Tang, S.X.; Tong, H.; Yanagishima, T.; Tanaka, H.; Tan, P. Morphology selection kinetics of crystallization in a sphere. *Nat. Phys.* **2021**, *17*, 121–127, doi:10.1038/s41567-020-0991-9.
2. Arai, S.; Tanaka, H. Surface-assisted single-crystal formation of charged colloids. *Nat. Phys.* **2017**, *13*, 503–509, doi:10.1038/nphys4034.
3. De Nijs, B.; Dussi, S.; Smallenburg, F.; Meeldijk, J.D.; Groenendijk, D.J.; Filion, L.; Imhof, A.; van Blaaderen, A.; Dijkstra, M. Entropy-driven formation of large icosahedral colloidal clusters by spherical confinement. *Nat. Mater.* **2015**, *14*, 56–60, doi:10.1038/nmat4072.
4. Irvine, W.T.M.; Vitelli, V.; Chaikin, P.M. Pleats in crystals on curved surfaces. *Nature* **2010**, *468*, 947–951, doi:10.1038/nature09620.
5. Tang, X.L.; Chen, W.; Li, L.B. The tough journey of polymer crystallization: Battling with chain flexibility and connectivity. *Macromolecules* **2019**, *52*, 3575–3591, doi:10.1021/acs.macromol.8b02725.
6. Agbolaghi, S.; Abbaspoor, S.; Abbasi, F. A comprehensive review on polymer single crystals-from fundamental concepts to applications. *Prog. Polym. Sci.* **2018**, *81*, 22–79, doi:10.1016/j.progpolymsci.2017.11.006.
7. Cheng, S.Z.D.; Lotz, B. Enthalpic and entropic origins of nucleation barriers during polymer crystallization: The hoffman-lauritzen theory and beyond. *Polymer* **2005**, *46*, 8662–8681, doi:10.1016/j.polymer.2005.03.125.
8. Strobl, G. Crystallization and melting of bulk polymers: New observations, conclusions and a thermodynamic scheme. *Prog. Polym. Sci.* **2006**, *31*, 398–442, doi:10.1016/j.progpolymsci.2006.01.001.
9. Luo, C.F.; Sommer, J.U. Frozen topology: Entanglements control nucleation and crystallization in polymers. *Phys. Rev. Lett.* **2014**, *112*, doi:10.1103/PhysRevLett.112.195702.
10. Luo, C.F.; Sommertt, J.U. Role of thermal history and entanglement related thickness selection in polymer crystallization. *ACS Macro Lett.* **2016**, *5*, 35–39, doi:10.1021/acsmacrolett.5b00668.
11. Li, C.Y. The rise of semicrystalline polymers and why are they still interesting. *Polymer* **2020**, *211*, doi:10.1016/j.polymer.2020.123150.
12. Alder, B.J.; Wainwright, T.E. Phase transition for a hard sphere system. *J. Chem. Phys.* **1957**, *27*, 1208–1209, doi:10.1063/1.1743957.
13. Rintoul, M.D.; Torquato, S. Metastability and crystallization in hard-sphere systems. *Phys. Rev. Lett.* **1996**, *77*, 4198–4201, doi:10.1103/PhysRevLett.77.4198.
14. Karayiannis, N.C.; Foteinopoulou, K.; Laso, M. Entropy-driven crystallization in dense systems of athermal chain molecules. *Phys. Rev. Lett.* **2009**, *103*, doi:10.1103/PhysRevLett.103.045703.
15. Karayiannis, N.C.; Foteinopoulou, K.; Laso, M. Twinning of polymer crystals suppressed by entropy. *Symmetry* **2014**, *6*, 758–780, doi:10.3390/sym6030758.
16. Karayiannis, N.C.; Foteinopoulou, K.; Laso, M. The role of bond tangency and bond gap in hard sphere crystallization of chains. *Soft Matter.* **2015**, *11*, 1688–1700, doi:10.1039/c4sm02707h.
17. Ni, R.; Dijkstra, M. Effect of bond length fluctuations on crystal nucleation of hard bead chains. *Soft Matter.* **2013**, *9*, 365–369, doi:10.1039/c2sm26969d.
18. Shakirov, T. Crystallisation in melts of short, semi-flexible hard-sphere polymer chains: The role of the non-bonded interaction range. *Entropy* **2019**, *21*, 856, doi:10.3390/e21090856.
19. Shakirov, T.; Paul, W. Crystallization in melts of short, semiflexible hard polymer chains: An interplay of entropies and dimensions. *Phys. Rev. E* **2018**, *97*, doi:10.1103/PhysRevE.97.042501.

20. Karayiannis, N.C.; Foteinopoulou, K.; Abrams, C.F.; Laso, M. Modeling of crystal nucleation and growth in athermal polymers: Self-assembly of layered nano-morphologies. *Soft Matter.* **2010**, *6*, 2160–2173, doi:10.1039/b923369e.
21. Karayiannis, N.C.; Foteinopoulou, K.; Laso, M. Spontaneous crystallization in athermal polymer packings. *Int. J. Mol. Sci.* **2013**, *14*, 332–358, doi:10.3390/ijms14010332.
22. Dietz, J.D.; Hoy, R.S. Two-stage athermal solidification of semiflexible polymers and fibers. *Soft Matter.* **2020**, *16*, 6206–6217, doi:10.1039/d0sm00754d.
23. Schilling, T.; Schope, H.J.; Oettel, M.; Opletal, G.; Snook, I. Precursor-mediated crystallization process in suspensions of hard spheres. *Phys. Rev. Lett.* **2010**, *105*, doi:10.1103/PhysRevLett.105.025701.
24. Kawasaki, T.; Tanaka, H. Formation of a crystal nucleus from liquid. *Proc. Natl. Acad. Sci. USA* **2010**, *107*, 14036–14041, doi:10.1073/pnas.1001040107.
25. Zaccarelli, E.; Valeriani, C.; Sanz, E.; Poon, W.C.K.; Cates, M.E.; Pusey, P.N. Crystallization of hard-sphere glasses. *Phys. Rev. Lett.* **2009**, *103*, doi:10.1103/PhysRevLett.103.135704.
26. Williams, S.R.; Royall, C.P.; Bryant, G. Crystallization of dense binary hard-sphere mixtures with marginal size ratio. *Phys. Rev. Lett.* **2008**, *100*, doi:10.1103/PhysRevLett.100.225502.
27. O'Malley, B.; Snook, I. Structure of hard-sphere fluid and precursor structures to crystallization. *J. Chem. Phys.* **2005**, *123*, doi:10.1063/1.1992475.
28. O'Malley, B.; Snook, I. Crystal nucleation in the hard sphere system. *Phys. Rev. Lett.* **2003**, *90*, doi:10.1103/PhysRevLett.90.085702.
29. Isobe, M.; Krauth, W. Hard-sphere melting and crystallization with event-chain monte carlo. *J. Chem. Phys.* **2015**, *143*, doi:10.1063/1.4929529.
30. Auer, S.; Frenkel, D. Line tension controls wall-induced crystal nucleation in hard-sphere colloids. *Phys. Rev. Lett.* **2003**, *91*, doi:10.1103/PhysRevLett.91.015703.
31. Van Blaaderen, A.; Ruel, R.; Wiltzius, P. Template-directed colloidal crystallization. *Nature* **1997**, *385*, 321–324, doi:10.1038/385321a0.
32. Chen, D.; Torquato, S. Confined disordered strictly jammed binary sphere packings. *Phys. Rev. E* **2015**, *92*, doi:10.1103/PhysRevE.92.062207.
33. Fortini, A.; Dijkstra, M. Phase behaviour of hard spheres confined between parallel hard plates: Manipulation of colloidal crystal structures by confinement. *J. Phys. Condens. Matter.* **2006**, *18*, L371–L378, doi:10.1088/0953-8984/18/28/l02.
34. Dijkstra, M.; van Roij, R.; Roth, R.; Fortini, A. Effect of many-body interactions on the bulk and interfacial phase behavior of a model colloid-polymer mixture. *Phys. Rev. E* **2006**, *73*, doi:10.1103/PhysRevE.73.041404.
35. Fortini, A.; Schmidt, M.; Dijkstra, M. Phase behavior and structure of model colloid-polymer mixtures confined between two parallel planar walls. *Phys. Rev. E* **2006**, *73*, doi:10.1103/PhysRevE.73.051502.
36. Benito, J.; Karayiannis, N.C.; Laso, M. Confined polymers as self-avoiding random walks on restricted lattices. *Polymers* **2018**, *10*, 1394, doi:10.3390/polym10121394.
37. Sandomirski, K.; Walta, S.; Dubbert, J.; Allahyarov, E.; Schofield, A.B.; Lowen, H.; Richtering, W.; Egelhaaf, S.U. Heterogeneous crystallization of hard and soft spheres near flat and curved walls. *Eur. Phys. J. Spec. Top.* **2014**, *223*, 439–454, doi:10.1140/epjst/e2014-02101-7.
38. Luo, C.F.; Kroger, M.; Sommer, J.U. Molecular dynamics simulations of polymer crystallization under confinement: Entanglement effect. *Polymer* **2017**, *109*, 71–84, doi:10.1016/j.polymer.2016.12.011.
39. Zhang, W.; Zou, L. Molecular dynamics simulations of crystal nucleation near interfaces in incompatible polymer blends. *Polymers* **2021**, *13*, 347.
40. Espinosa, J.R.; Vega, C.; Valeriani, C.; Frenkel, D.; Sanz, E. Heterogeneous versus homogeneous crystal nucleation of hard spheres. *Soft Matter.* **2019**, *15*, 9625–9631, doi:10.1039/c9sm01142k.
41. Curk, T.; de Hoogh, A.; Martinez-Veracoechea, F.J.; Eiser, E.; Frenkel, D.; Dobnikar, J.; Leunissen, M.E. Layering, freezing, and re-entrant melting of hard spheres in soft confinement. *Phys. Rev. E* **2012**, *85*, doi:10.1103/PhysRevE.85.021502.
42. Soik, S.M.; Sharp, T.A. Effects of spherical confinement and backbone stiffness on flexible polymer jamming. *Phys. Rev. E* **2019**, *99*, doi:10.1103/PhysRevE.99.052505.
43. Meldrum, F.C.; O'Shaughnessy, C. Crystallization in confinement. *Adv. Mater.* **2020**, *32*, doi:10.1002/adma.202001068.
44. Bechinger, C. Colloidal suspensions in confined geometries. *Curr. Opin. Colloid Interface Sci.* **2002**, *7*, 204–209, doi:10.1016/s1359-0294(02)00055-9.
45. Pusey, P.N.; Vanmegen, W. Phase-behavior of concentrated suspensions of nearly hard colloidal spheres. *Nature* **1986**, *320*, 340–342, doi:10.1038/320340a0.
46. Neser, S.; Bechinger, C.; Leiderer, P.; Palberg, T. Finite-size effects on the closest packing of hard spheres. *Phys. Rev. Lett.* **1997**, *79*, 2348–2351, doi:10.1103/PhysRevLett.79.2348.
47. Pieranski, P.; Strzelecki, L.; Pansu, B. Thin colloidal crystals. *Phys. Rev. Lett.* **1983**, *50*, 900–903, doi:10.1103/PhysRevLett.50.900.
48. Ramos, P.M.; Karayiannis, N.C.; Laso, M. Off-lattice simulation algorithms for athermal chain molecules under extreme confinement. *J. Comput. Phys.* **2018**, *375*, 918–934, doi:10.1016/j.jcp.2018.08.052.
49. Karayiannis, N.C.; Laso, M. Monte carlo scheme for generation and relaxation of dense and nearly jammed random structures of freely jointed hard-sphere chains. *Macromolecules* **2008**, *41*, 1537–1551, doi:10.1021/ma702264u.
50. Karayiannis, N.C.; Foteinopoulou, K.; Laso, M. The characteristic crystallographic element norm: A descriptor of local structure in atomistic and particulate systems. *J. Chem. Phys.* **2009**, *130*, doi:10.1063/1.3077294.

51. Ramos, P.M.; Herranz, M.; Foteinopoulou, K.; Karayiannis, N.C.; Laso, M. Identification of local structure in 2-d and 3-d atomic systems through crystallographic analysis. *Crystals* **2020**, *10*, 1008, doi:10.3390/cryst10111008.
52. Herranz, M.; Santiago, M.; Foteinopoulou, K.; Karayiannis, N.C.; Laso, M. Crystal, fivefold and glass formation in clusters of polymers interacting with the square well potential. *Polymers* **2020**, *12*, 1111, doi:10.3390/polym12051111.
53. Pant, P.V.K.; Theodorou, D.N. Variable connectivity method for the atomistic monte-carlo simulation of polydisperse polymer melts. *Macromolecules* **1995**, *28*, 7224–7234, doi:10.1021/ma00125a027.
54. Mavrantzas, V.G.; Boone, T.D.; Zervopoulou, E.; Theodorou, D.N. End-bridging monte carlo: A fast algorithm for atomistic simulation of condensed phases of long polymer chains. *Macromolecules* **1999**, *32*, 5072–5096, doi:10.1021/ma981745g.
55. Karayiannis, N.C.; Malshe, R.; Kroger, M.; de Pablo, J.J.; Laso, M. Evolution of fivefold local symmetry during crystal nucleation and growth in dense hard-sphere packings. *Soft Matter.* **2012**, *8*, 844–858, doi:10.1039/c1sm06540h.
56. Karayiannis, N.C.; Malshe, R.; de Pablo, J.J.; Laso, M. Fivefold symmetry as an inhibitor to hard-sphere crystallization. *Phys. Rev. E* **2011**, *83*, doi:10.1103/PhysRevE.83.061505.
57. Humphrey, W.; Dalke, A.; Schulten, K. Vmd: Visual molecular dynamics. *J. Mol. Graphics Modell.* **1996**, *14*, 33–38, doi:10.1016/0263-7855(96)00018-5.
58. Hoy, R.S.; Karayiannis, N.C. Simple model for chain packing and crystallization of soft colloidal polymers. *Phys. Rev. E* **2013**, *88*, doi:10.1103/PhysRevE.88.012601.
59. Nguyen, H.T.; Smith, T.B.; Hoy, R.S.; Karayiannis, N.C. Effect of chain stiffness on the competition between crystallization and glass-formation in model unentangled polymers. *J. Chem. Phys.* **2015**, *143*, doi:10.1063/1.4932193.
60. Kumar, S.K.; Vacatello, M.; Yoon, D.Y. Off-lattice monte-carlo simulations of polymer melts confined between 2 plates .2. Effects of chain-length and plate separation. *Macromolecules* **1990**, *23*, 2189–2197, doi:10.1021/ma00210a012.
61. Kumar, S.K.; Vacatello, M.; Yoon, D.Y. Off-lattice monte-carlo simulations of polymer melts confined between 2 plates. *J. Chem. Phys.* **1988**, *89*, 5206–5215, doi:10.1063/1.455611.
